# Batch-to-Batch Variation in Laser-Inscribed Graphene (LIG) Electrodes for Electrochemical Sensing

**DOI:** 10.3390/mi15070874

**Published:** 2024-06-30

**Authors:** Yifan Tang, Geisianny A. Moreira, Diana Vanegas, Shoumen P. A. Datta, Eric S. McLamore

**Affiliations:** 1 Department of Plant and Environmental Sciences, Clemson University, Clemson, SC 29631, USA; ytang95@jh.edu; 2 Department of Agricultural Sciences, Clemson University, Clemson, SC 29631, USA; gamm.bio@gmail.com; 3 Department of Environmental Engineering and Earth Sciences, Clemson University, Clemson, SC 29634, USA; dvanega@clemson.edu; 4 Department of Mechanical Engineering, MIT Auto-ID Labs, Massachusetts Institute of Technology, Cambridge, MA 02139, USA; shoumen@mit.edu; 5 Biomedical Engineering Program, Medical Device (MDPnP) Interoperability and Cybersecurity Labs, Department of Anesthesiology, Massachusetts General Hospital, Harvard Medical School, Cambridge, MA 02139, USA

**Keywords:** laser-inscribed graphene, LIG, sensor, batch, variation, scalability, manufacturing

## Abstract

Laser-inscribed graphene (LIG) is an emerging material for micro-electronic applications and is being used to develop supercapacitors, soft actuators, triboelectric generators, and sensors. The fabrication technique is simple, yet the batch-to-batch variation of LIG quality is not well documented in the literature. In this study, we conduct experiments to characterize batch-to-batch variation in the manufacturing of LIG electrodes for applications in electrochemical sensing. Numerous batches of 36 LIG electrodes were synthesized using a CO_2_ laser system on polyimide film. The LIG material was characterized using goniometry, stereomicroscopy, open circuit potentiometry, and cyclic voltammetry. Hydrophobicity and electrochemical screening (cyclic voltammetry) indicate that LIG electrode batch-to-batch variation is less than 5% when using a commercial reference and counter electrode. Metallization of LIG led to a significant increase in peak current and specific capacitance (area between anodic/cathodic curve). However, batch-to-batch variation increased to approximately 30%. Two different platinum electrodeposition techniques were studied, including galvanostatic and frequency-modulated electrodeposition. The study shows that formation of metallized LIG electrodes with high specific capacitance and peak current may come at the expense of high batch variability. This design tradeoff has not been discussed in the literature and is an important consideration if scaling sensor designs for mass use is desired. This study provides important insight into the variation of LIG material properties for scalable development of LIG sensors. Additional studies are needed to understand the underlying mechanism(s) of this variability so that strategies to improve the repeatability may be developed for improving quality control. The dataset from this study is available via an open access repository.

## 1. Introduction

Laser-inscribed graphene (LIG) is an emerging material that was originally discovered by the Tour group [1]. LIG is created by irradiating a carbon source (in most cases polyimide films) with a laser to graphitize sp^3^ carbon to sp^2^-hybridized carbon (see Supplemental Figure S1) [2]. Use of LIG as a technology platform in electronics has grown exponentially in the last decade [3]. Compared to other methods for conductive carbon film synthesis, laser ablation is one of the most efficient techniques used for device fabrication [4], which is rooted in the excellent intrinsic properties of LIG and the ability to customize numerous micro-device features. 

LIG is an attractive platform for micro-device fabrication, as the one-step synthesis is relatively simple and requires a single instrument (most often a CO_2_ laser-writing system). Examples of LIG-based micro-devices include micro-supercapacitors [5], soft actuators [6], triboelectric generators [7], gas sensors [8], strain/motion sensors [9,10], saliva sample tubes with embedded biosensors [11,12,13], portable food safety sensors [14], and flexible soil sensors for column studies [15], among others. In sensing and biosensing applications, the particular properties of interest are high specific surface area, relatively high electrical conductivity, ability to micropattern custom geometries, and flexibility [16,17].

Microscale sensors have been developed based on LIG for targeting ions [18,19], sugars [20,21], biomolecules [22,23], bacteria [24], and viruses [12,13,25]. One of the major limitations for developing micro-sensors based on LIG is the unknown scalability of the fabrication process. Modern CO_2_ laser-writing systems have a wide range of control features (e.g., rastering, power density, speed). Behrent et al. [26] conducted an excellent study on the optimum laser control settings during fabrication of LIG, specifically focusing on electrochemical sensor/biosensor design (this manuscript also addresses key operational protocols such as cool-down time, etc.). This detailed study investigated numerous aspects beyond laser equipment settings, including direction of patterning, and to some extent patterning width.

Conceptually, many devices can be micro-patterned using common computer-aided design (CAD) software and applying the optimum laser control settings [26,27,28]. However, polyimide films are known to undergo deformation during the graphitization process (e.g., local bending), which alters the focal plane of the laser when printing large arrays. For the fabrication of relatively small laser processing jobs (e.g., three replicate LIG electrodes to be used in sensor development), the issue of film deformation is less problematic, but electrode-to-electrode variation exists. In addition to local deformation during graphitization, analysis by electron and Raman microscopy indicate that the surface chemistry and feature size are highly variable [14,15,26,28]. For this reason, studies of the batch-to-batch variation in LIG are critical for developing quality control (QC) practices.

Raman spectroscopy and nanoscale metrology [29,30] have been used for QC screening of graphene materials (primarily powders). However, these methods were not applied to graphene films on flat 2D surfaces. Recently, Qian et al. [25] developed a quality control screening framework and data analysis technique (hierarchical clustering) for quality control studies of LIG using cyclic voltammetry. However, batch-to-batch variation in LIG fabrication has not been characterized in detail and the source of the variation is largely unknown. Thus, it is challenging to perform detailed life cycle studies or predict scalability of LIG micro-sensor technologies at the scale of competitive devices based on classic manufacturing (e.g., the silicon semiconductor industry).

In this manuscript, we conduct the first detailed study of the batch-to-batch variation in LIG electrodes fabricated with a CO_2_ laser and polyimide film. We apply some of the most common techniques employed in sensor research labs (electrochemical analysis, goniometry, and stereomicroscopy). We report LIG hydrophobicity and electrochemical behavior in batches of at least 36 electrodes. Additionally, we characterize batches of LIG metallized with platinum (Pt) to study whether electrodeposition may affect the electrochemical performance of LIG batches. 

## 2. Materials and Methods

### 2.1. Chemicals and Reagents

Potassium ferrocyanide (K_4_Fe(CN)_6_), potassium ferricyanide (K_3_Fe(CN)_6_), potassium chloride (KCl), lead acetate (30% *w*/*v*), morpholinoethanesulfonic acid hydrate (MES), tris(Hydroxymethyl)aminomethane (Tris), and 4-(2-Hydroxyethyl)-1-piperazine ethanesulfonic acid (HEPES) were purchased from Fisher Scientific (Pittsburgh, PA, USA). Isotonic bicarbonate buffer (213 mM-NaCl + 65 mM-NaHCO_3_) was purchased from NeilMed (Santa Rosa, CA, USA). Chloroplatinic acid (8 *w*/*v*) was purchased from Sigma-Aldrich (St Louis, MO, USA). Silver/silver chloride (Ag/AgCl) reference electrode and Pt-wire counter electrode were purchased from BASi (West Lafayette, IN, USA). Polyimide film (electrical grade polyimide film, type HN, 0.0050″ thick) and chemical-resistant polyvinyl chloride sheets (1/16″ thick, 12 × 12 sheet) were obtained from McMaster-Carr (Elmhurst, IL, USA). Metal alloy tape was purchased from Beijing Electronics Store (Beijing, China). Lacquer for electrode passivation was purchased from a local grocer in Clemson, SC. Female USB2.0 (Type A) port socket connectors and insulated 28 AWG jumper wires were purchased from Amazon. NeilMed saline rinse powder (an isotonic bicarbonate buffer) and double-sided Scotch tape were purchased from a local pharmacy.

### 2.2. Laser-Inscribed Graphene Electrode Fabrication

Details and step-by-step methods for LIG fabrication are described in McLamore et al. [31]. Briefly, electrode patterns were first designed in CorelDraw (Corel Corporation, Ottawa, ON, Canada; see Supplemental Figure S1B,C). Patterned electrodes were graphitized on polyimide film using a Universal CO_2_ laser system (version VLS3.60, Scottsdale, AZ, USA). The heat map by Behrent et al. [26] was reproduced, with nearly identical results (see Supplemental Figure S2). Within a select range, additional optimization was conducted to ensure proper laser scribing in our instrument. Based on this study, the following settings were used: 5.8 cm distance from lens to polyimide surface, Z axis offset of 0.005″, image density of 7, rastering speed of 75%, power of 40%, and density of 1000 PPI. After graphitization, LIG electrodes were immediately rinsed with 70% ethanol thrice, and then rinsed with DI water. Fast drying poly-gel acrylic lacquer was used to passivate the electrode(s), and then bonding pads (0.13 mm thick conductive polyester metal tape) were pressed with forceps. Depending on the type of experiment, a single (working) LIG electrode or three-electrode LIG system was patterned and graphitized.

A USB plug-and-play microdevice was fabricated for connecting the three-electrode LIG system (referred to as the “sensor chip”) to the potentiostat. Details are described in the protocol by Casso-Hartmann et al. [11]. Briefly, two jumper wires were soldered to the outside pins of a female USB-A plug. Next, the two inner pins were jumped using solder, and a third wire was soldered to this pin junction. These three jumper wires were the connection point for the reference, counter, and working electrodes, respectively. The USB-A plastic case was attached and pressed firmly using pliers. The LIG sensor chip geometry was designed in CorelDraw such that the bonding pad of each of the three electrodes aligns with these USB-A pins. Strips (0.3 cm by 3.5 cm) of chemical-resistant 1/16″ thick PVC were cut as the electrode base. After graphitization of the LIG three-electrode system, double-sided tape was applied to the back of the LIG flexible chip and then fixed to the PVC base. This micro-device (PVC backing with LIG sensor chip adhered) directly plugs to the USB-A female port socket, and jumper wires were connected to the PalmSens potentiostat.

### 2.3. Nanoplatinum (nPt) Electrodeposition on LIG Electrodes

For electrodeposition of nanoplatinum (nPt) on LIG single electrode or LIG sensor chip (working electrode), the stem was passivated and dried at room temperature as described above. The LIG working electrode and a Pt wire (99.9%) were immersed into a solution containing 0.728% chloroplatinic acid and 0.002% lead acetate. For the sensor chip, only the LIG working electrode was connected to the power supply. The LIG working electrode and the Pt wire were connected to a power supply (either DC or AC power supply). For galvanostatic deposition (GED), a DC power supply was used (Tektronix, Beaverton, OR, USA). GED was tested at 10 V for a plating time of either 90 s or 180 s based on previous work [13,32]. We also tested a frequency-modulated deposition (FMED) technique using an AC waveform generator (SDG2042X Arbitrary Waveform Function-Generators, Siglent, OH, USA). Two different FMED waveforms (0.5 Hz) were tested: (i) 5 V_DC_ with an amplitude of 10 V_AC_ (peak to peak), and (ii) 8 V_DC_ with an amplitude of 4 V_AC_ (peak to peak). Total plating times of 90 s and 180 s were tested at both FMED waveform settings. In all cases, the maximum potential was 10 V. Details of the waveforms are described by Tang et al. [33]. After electrodeposition of nPt, electrodes were rinsed with DI water gently prior to testing.

### 2.4. Goniometry

Hydrophobicity was analyzed using a Droplet lab DROPOMETER-M (Markham, ON, Canada). After LIG fabrication and preparation, electrodes were stabilized and beveled on the testing instrument platform using the sample mounts. A 2 µL aliquot of sample was carefully pipetted on the working area of LIG electrode. The following solutions were tested: DI water, MES buffer, Tris buffer, HEPES buffer, 2 × isotonic bicarbonate buffer, platinum plating solution, and ferri/ferrocyanide solution. 

Contact angle calculations were based on the polynomial method (non-axisymmetric drop). The instrument camera was used to capture a static picture in sessile droplet mode [34]. In the software, key features were identified in the images (e.g., edges as drop region profile of interest) following manufacturer recommendations [35]. In preliminary analysis, Young–Laplace fitting (axisymmetric) methods showed significant errors for the LIG surface, up to 60% for the same sample tested multiple times sequentially. Thus, non-axisymmetric drop was used throughout.

An experiment was performed to determine the batch-to-batch variation of LIG hydrophobicity (contact angle). Four unique batches (nine electrodes each) were prepared on individual days, by the same operator. The contact angle was measured for each batch of nine electrodes using the non-axisymmetric drop method. Contact angle was calculated by Droplet lab software (version 1.4.0.10), and images of each test were archived.

### 2.5. Electrochemical Analysis

All pH measurements were conducted with a Thermo Orion A211 Benchtop pH Meter and calibration standards (Waltham, MA, USA). Electrochemical characterization of LIG, LIG-biochip, nPt-LIG, and nPt-biochip were carried out using a Multi PalmSens4 potentiostat (PalmSens BV, GA, Houten, The Netherlands). Where batches are referred to, six unique batches (six electrodes each) were prepared on individual days, by the same operator, for each electrode type.

Open circuit potential (OCP) and cyclic voltammetry (CV) with ferri/ferroocyanide as a redox probe were analyzed. All tests were conducted in BASi electrochemical glass cells. Single LIG electrode studies used Ag/AgCl as the reference electrode and a Pt-wire as the counter electrode. Sensor chip studies used LIG as the working and counter electrodes, with metal alloy tape mounted on LIG as the reference electrode. All CV experiments were conducted at room temperature with stationary electrodes in 2.5 mM potassium ferricyanide and 2.5 mM potassium ferrocyanide, with 100 mM potassium chloride as the electrolyte. Where noted, non-Faradaic OCP testing was conducted in bicarbonate buffer (i.e., no redox probe). Bicarbonate buffer is commonly used in biological sensing (e.g., in isotonic preparations for nasal swabs) [12,13]. For OCP measurements, recording time was 120 s and data acquisition rate was 10 kHz. All testing was conducted in bicarbonate buffer as described by Moreira et al. [12,13].

In some experiments, conditioning experiments utilized ten successive CV sweeps at a scan rate of 200 mV/s over the range of −0.8 V to 0.8 V. Details of the experimental methods are shown in the published protocol by Tang et al. [36]. Oxidation peak (i_op_) and area between anodic and cathodic curve (ABC) were calculated from CV curves according to Qian et al. [25]. Peak oxidation current was defined as the maximum current within the oxidation window of ferrocyanide based on previous research. C_p_ was calculated using the approach by Wang et al. [37] after determination of scan rates that did not induce significant Ohmic drop. Unless otherwise noted, six batches of LIG electrodes were tested (six electrodes in each batch; n(total) = 36).

### 2.6. Statistical Analysis

Data analyses were conducted by R studio (version 1.1.463). All variables were tested for normality using the Shapiro test. Significance was tested within groups using a *t*-test or Wilcox test as noted. To compare differences across groups, the least significant difference (LSD) test was used. All statistical tests are reported at an α level of 0.05.

## 3. Results and Discussion

Laser settings for the fabrication of LIG were optimized based on the approach by Behrent et al. [26] (Supplemental Figure S2). A heat map was created for selecting optimum laser settings using a scoring system. Results from three analyses were used to develop an equivalent weight scoring system: (i) stereomicroscopy examination (pass/fail), based on Behrent et al. [26]; (ii) peak oxidative current (higher than 150 μA), according to Qian et al. [25]; and (iii) area between anodic/cathodic sweep (more than 100 μV-A). Based on this equivalent weight scoring system, the optimum settings for LIG fabrication were determined to be 40% power, 75% speed, and 1000 lines per inch (raster mode), with a material thickness (Z_axis_) of 0.005”. Additional details on the scoring system for optimizing laser settings are described in Tang [33]. Protocols with details of optimal manufacture settings are available on Protocol I/O [11,31]. 

### 3.1. Goniometry

**Figure 1:**
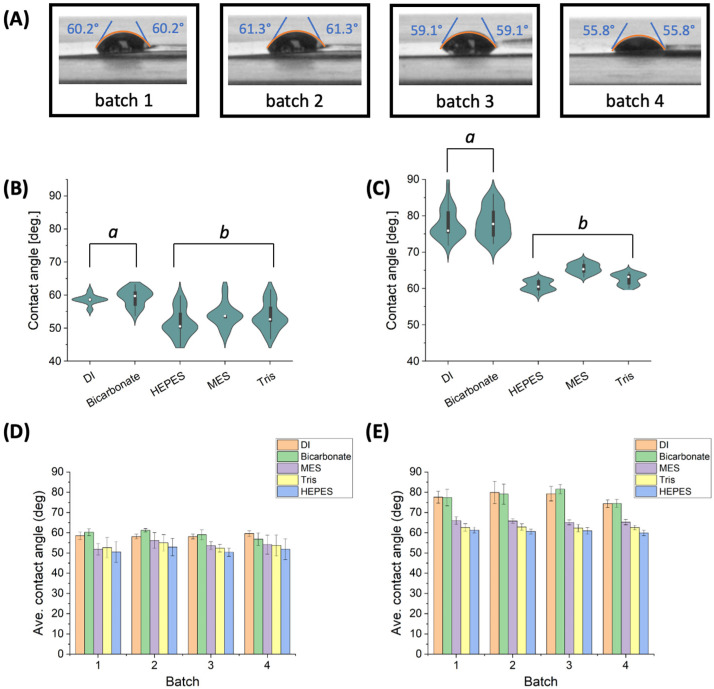
Hydrophobicity study of LIG and nPt-LIG. (**A**) Representative images from goniometry testing of four electrode batches (LIG sample with 2 μL DI). Results of non-axisymmetric method and calculated contact angle shown on each image. Violin plots show contact angle in testing liquids for: (**B**) non-modified LIG electrodes, and (**C**) nPt-LIG electrodes. White dots represent median value, black boxes show range from the lower to the upper quartile, whiskers present the variability outside upper and lower quantile, and the shape of violin indicates the data density (*n* = 24 for each group). Average contact angle is shown for: (**D**) non-modified LIG, and (**E**) nPt-LIG electrodes. Error bars represent standard deviation (n = 6 electrodes in each batch). Lowercase letters (a,b) indicate group subsetting based on LSD, where the same letter indicates no significant different between groups (α = 0.05).

Batch-to-batch variation of LIG hydrophobicity immediately after graphitization was analyzed for four unique batches of single LIG electrodes and nPt-LIG (six electrodes each). The average contact angle for LIG in DI (58.6 ± 1.4°) indicates that LIG has a hydrophilic surface under these conditions, which is similar to the results reported in the detailed study by Hjort et al. [19]. Representative images of droplets are shown in Figure 1A. Isotonic bicarbonate buffer supplemented with sodium chloride is a common testing solution for LIG biosensors [12,13,25]. The mean contact angle in 2 × isotonic buffer (59.3 ± 2.6°) was not significantly different than DI, which is expected (Figure 1B). The contact angle of non-modified LIG varied by less than 5% in DI and isotonic bicarbonate (within fabrication batches; see Figure 1D). In addition to testing the simple bicarbonate buffer, three other common biological buffers were screened, including compounds that have surfactant-like properties. Morpholinoethanesulfonic acid hydrate (MES) and 4-(2-Hydroxyethyl)-1-piperazine ethanesulfonic acid (HEPES) are organosulfonic acids with zwitterionic behavior. Tris(Hydroxymethyl)aminomethane (Tris) is a primary amino compound and an emulsifying agent. Figure 1B shows that the HEPES, MES, and Tris contact angle is significantly lower than DI, indicating that each behaves as a mild surfactant, impacting surface tension. The contact angle varied by 6% to 8% within these groups, which is higher than DI/bicarbonate solutions. 

Preliminary experiments indicated that the laser system may be limited in terms of the number of electrodes that can be fabricated in one day. When a batch of 36 electrodes was fabricated in a single day, the contact angle varied by more than 30%. Thus, we fabricated and analyzed 36 electrodes by creating four batches of nine electrodes each, with a 30 min laser downtime between each batch. Additional studies are required to identify the source of the variation during the LIG manufacturing process, but the reduced variation (from 30% to less than 5%) indicates that future manufacturing protocols should consider laser maintenance (e.g., lens cleaning), operational frequency, and batch size as important control factors. 

After metallization with nPt, the contact angle in DI and isotonic carbonate buffer (78 ± 4°) increased by nearly 20%, indicating that the surface is more hydrophobic (Figure 1C). The mean contact angle in 2 × isotonic buffer was nearly identical in these two solutions and varied by 5% within fabrication batches (Figure 1E). The contact angle in the HEPES, MES, and Tris buffers was similar (63 ± 2°, which is higher than all LIG experiments). These data are important for understanding the interfacial phenomena in different buffers, particularly when zwitterionic molecules are present in testing solutions [38]. 

### 3.2. Electrochemical Characterization

To further explore the behavior of LIG electrodes, single working electrode and three-electrode non-modified LIG were tested. Open circuit potential (OCP) and cyclic voltammetry (CV) were analyzed at room temperature. For Faradaic testing, ferricyanide/ferrocyanide (2.5 mM each) was used as the redox probe couple, and potassium chloride (100 mM) as the electrolyte.

OCP (non-Faradaic chronopotentiometry) was measured in 2 × sodium bicarbonate isotonic buffer (pH = 7.6). The average OCP for single LIG electrodes (209 ± 11 mV) varied by less than 5% and was more than two times higher than LIG sensor chips (90.2 ± 8.8 mV). This experiment was repeated in an equimolar mixture of potassium ferri/ferrocyanide (2.5 mM each) to determine the half-cell constant (E_0_). The average E_0_ value for LIG electrodes was 93.5 ± 13.9 mV. See Supplemental Figure S3 for details on all solutions tested.

After characterizing OCP in multiple solutions, a ferri/ferrocyanide solution was used to study current response during CV (pH 6.8). Since both LIG and the ferri/ferrocyanide redox couple have negative charge under these testing conditions, it is likely that some electrical repulsion may have occurred at the LIG surface. One common technique to alleviate this problem is use of repetitive CV sweeps (i.e., electro-conditioning). This is a known mechanism for stabilizing the dielectric layer in electrochemical sensors and is often used in sensor development [39]. Thus, batches of 36 LIG electrodes were tested using ten successive CV sweeps for both single-electrode LIG and LIG sensor chips. Oxidation peak current (i_op_) data were extracted from CV curves as the response variable. 

**Figure 2:**
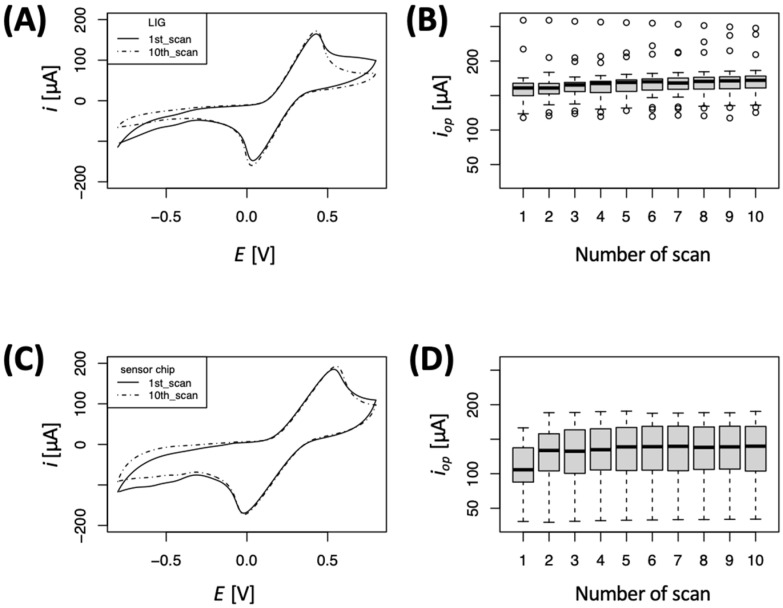
Cyclic voltammetry at 200 mV/s for LIG working electrodes (ferro/ferricyanide redox couple). Representative CV plots for initial (1st) and final (10th) electro-conditioning scan, and box plots showing peak oxidative current; n = 36 electrodes for each experiment. In box plots, dashed lines represent data range and open circles represent outliers (values outside the range of ±1.5 times interquartile range). (**A**) Representative CV of single LIG electrode, (**B**) boxplot of single LIG peak current, (**C**) representative CV of LIG sensor chip, and (**D**) boxplot of LIG sensor chip peak current.

Figure 2 shows representative cyclic voltammograms at 200 mV/s for LIG working electrodes using 2.5 mM potassium ferrocyanide and 2.5 mM ferricyanide as redox probes, with 100 mM potassium chloride as electrolyte. Figure 2A,C shows representative CV plots for the initial (1st scan) and final (10th scan) scan during LIG electro-conditioning. These voltammograms visualize the change in peak anodic/cathodic current during electro-conditioning, and slight changes in the archetypical shape of the CV. The oxidation and reduction peaks were more defined for the single LIG electrode than the LIG sensor chip assembly. Peak separation (ΔE_p_) for the single LIG electrode system was 205 ± 50 mV, which indicates relatively slow electron transfer kinetics under these testing conditions. For the LIG sensor chip the ΔE_p_ was nearly double (519 ± 20 mV). Figure 2B,D show box plots for 36 replicates for the single LIG and LIG sensor chip, respectively, and visualize the mean, standard deviation, interquartile range, and outliers for each electrode type. The mean i_op_ for the single LIG electrode was higher than the LIG sensor chip, which is expected since LIG was used as the counter electrode in the chip design (compared to 99.9% Pt wire used in single LIG electrode assembly). Although the LIG sensor chip has a relatively low anodic peak current under these conditions, this device represents a portable test as compared to the single LIG electrode system, which is a laboratory testing setup. 

To test for normality, CV data were further analyzed using a Shapiro–Wilk test (α = 0.05). The results indicate that single LIG assembly peak oxidative current was not normally distributed in electro-conditioning experiments. Conversely, data for the LIG sensor chip assembly were normally distributed during electro-conditioning. Thus, different methods were chosen accordingly to test for significant differences amongst conditioning scans. A Wilcox test was used for the single LIG assembly (non-normal) and a two-tailed *t*-test was used for nPt-LIG single electrodes. Supplemental Figure S4 shows a heat map of the difference testing results for each LIG electrode assembly. The dark grey shading indicates *p*-values smaller than 0.05 (significant difference in peak oxidation current), while the light grey shading represents *p*-values larger than 0.05 (no significant difference). The single LIG assembly requires at least four successive conditioning scans for the peak oxidative current to stabilize at 189 ± 20 μA. For the LIG sensor chip, only two scans were required to stabilize the dielectric layer at 155 ± 25 μA.

### 3.3. Nanoplatinum Deposition on LIG Electrodes

**Figure 3:**
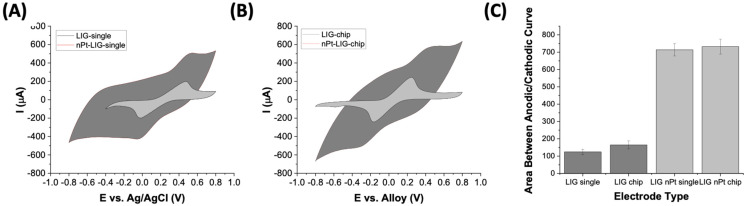
Representative CV plots of LIG and nPt-LIG in ferri/ferrocyanide at 200 mV/s (100 mM potassium chloride as electrolyte). (**A**) Single LIG electrodes (non-modified and after nPt coating). A Pt wire was used as counter electrode, and Ag/AgCl as reference electrode. (**B**) LIG sensor chip (non-modified and after nPt coating). Metal alloy tape was used as reference electrode, and LIG as counter electrode. (**C**) Area between anodic/cathodic sweep (in μV-A) for LIG and nPt-LIG in each assembly. At least 50 electrodes were tested for each electrode type (details in Supplemental Tables S2 and S3).

As most LIG electrodes are metallized for sensor applications [13,14,15,19,24], we tested the electrochemical behavior of LIG plated with nanoplatinum (nPt) in both the single LIG electrode and the LIG sensor chip format. After electro-conditioning the working electrode, LIG was immediately transferred to a chloroplatinic acid/lead acetate solution (pH 1.2) for electrodeposition of nPt. Figure 3 shows representative CV scans for LIG and nPt-LIG single-electrode and sensor chip assemblies. The i_op_ was 150~250 μA higher after nPt deposition, and the peak was more pronounced for the single LIG electrode assembly (Figure 3A,B). The ΔEp for both the single nPt-LIG electrodes (143 ± 3 mV) and nPt-LIG sensor chip (403 ± 43 mV) decreased significantly after metallization, which indicates an increase in electron transfer kinetics, as expected. However, the data indicate that the electron transfer properties under these conditions are relatively slow. The ABC (a proxy of specific capacitance) was seven times as high after nPt deposition, indicating a significant increase in surface charge (capacitance) for the metal–carbon hybrid material. The observed increase in capacitance (and peak current) has been shown with many other types of carbon electrodes [40,41], including LIG [12,13,14]. Raw CV data are presented in Supplemental Figure S5 and available in the Zenodo repository (see data availability statement). 

### 3.4. Batch-to-Batch Variation of Anodic Peak Current

**Figure 4:**
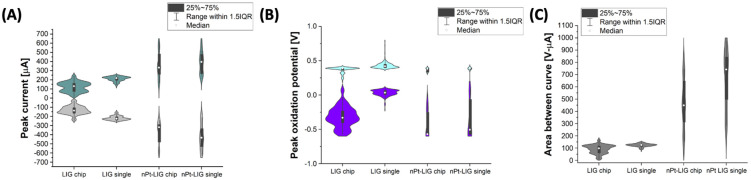
Average peak oxidation and reduction for LIG assemblies: (**A**) peak oxidation current (aqua violins) and reduction current (grey violins); (**B**) peak oxidation potential (teal violins) and reduction potential (purple violins); and (**C**) area between curve (anodic-cathodic sweep). All scans conducted at 25 °C in ferri/ferrocyanide solution at 200 mV/s (100 mM potassium chloride as electrolyte). White dots represent median value, black boxes range from the lower to the upper quartile, whiskers present the variability outside upper and lower quantile, and the shape of violin plot indicates the data density.

The experiment in Figure 3 was repeated in four batches of 36 electrodes as described previously. Four individual sets of each electrode type were fabricated on a different day by the same operator. Violin plots (Figure 4) indicate that the LIG sensor chip assembly had lower oxidation and reduction peaks than the LIG, with more noise (approximately 30% variation). However, when the working electrode was metallized with nPt, the effect absolves, and there is not a statistically significant difference between the single LIG and LIG sensor chip (oxidative and reductive peaks). Electron micrographs from our previous work with this material indicate that nPt coats the LIG surface. These results indicate that electrodeposition of nPt on LIG homogenizes the surface charge and dielectric layer. The peak oxidation potential (Figure 4B) did significantly change for any assembly (385 ± 31 mV). However, the peak reduction potential for single LIG (31 ± 45 mV) was significantly lower than the other electrode assemblies (362 ± 202 mV). Further study is required to understand the variation in oxide groups on LIG before and after metal deposition, which may provide insight into the variation in E_rp_. Individual plots of each dataset are shown in Supplemental Figure S6.

The least significant difference (LSD) test was conducted in R studio for statistical analysis [42]. Supplemental Figure S6 shows the results of CV analysis (i_op_ and ABC were calculated from each CV experiment). For all batches, Pt electrodeposition significantly increased i_op_ and ABC, similar to the analysis of all merged batches in Figure 4. The specific type of Pt electrodeposition had a significant impact on the peak current and potential in all batches, which illuminates the need for additional studies focused on nanometal characterization in LIG systems. When analyzed individually, a distinct grouping of I_op_ for each of the batches was observed, which explains the bimodal distributions in Figure 4. For this reason, the clustering analysis tool developed by Qian et al. is critical to maximize performance (especially if CV or other DC potential techniques are used for signal transduction). 

**Figure 5:**
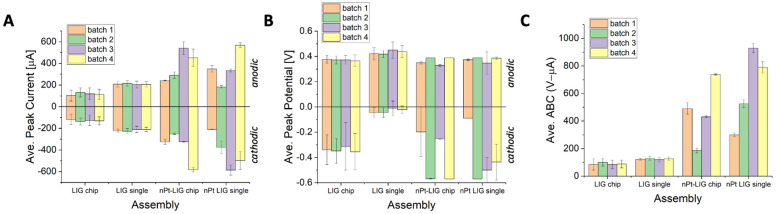
Average electrochemical parameters during batch testing. Each batch consists of six replicate electrodes. (**A**) Average peak oxidation (anodic) and reduction (cathodic) current; (**B**) peak oxidation potential (teal violins) and reduction potential (purple violins); and (**C**) area between curve (anodic-cathodic sweep). All scans conducted at 25 °C in ferri/ferrocyanide solution at 200 mV/s (100 mM potassium chloride as electrolyte). Error bars represent standard deviation of the arithmetic mean (n = 6 in each batch).

Figure 5 shows the average batch-to-batch variation based on CV. Using oxidative peak as the response variable, the LIG single electrode had the lowest variability (less than 2% variation across batches), and the LIG sensor chip was four times higher (8% variation across batches). This was expected, as the use of a commercial counter and reference electrode is more efficient than custom designs. However, the sensor chip design is applicable for point-of-need sensing, while the single LIG electrode system is only valid for controlled lab studies. The variation within batches was approximately 10% for both LIG single-electrode and sensor chip assemblies. As shown in Figure 5B, the trend in batch variation using cathodic (reductive) peak current as the response variable was similar, but the variation within batches (12–15%) and across batches (11%) was higher than the anodic sweep. Likewise, the variation for ABC (Figure 5C) was even higher (15% across batches and 12% within batches). The peak potential varied by 5% within groups, and 2% across groups (result consistent for either anodic or cathodic sweep). The variation for the LIG sensor chip was slightly higher (5–15%), although the mean current and ABC were lower. 

The batch variation for nPt-LIG was higher than the LIG single electrode, although the mean values were similarly significantly improved relative to the LIG electrode (without nPt). There was no significant difference between the variability of the nPt-LIG single electrode and the nPt-LIG sensor chip, which is an important design improvement compared to bare LIG electrodes. The most pronounced variability for nPt-LIG was the ABC (Figure 5C). After metallization, the batch variation was 15–20% within batches and 30–40% across batches. Supplemental Figure S6 shows that this result is likely due to the method of metal deposition. Galvanostatic electrodeposition led to a lower peak current than frequency-modulated electrodeposition (AC waveform) and had less batch variation (both within and across batches). The noted increase in capacitive behavior is critical for many sensors [12,13,43,44,45], but may come at the expense of high batch variation when scaling sensors (using the methods herein). While electrodeposition is a simple and reliable technique, precise control over the microscale structuring is extremely challenging. Techniques such as simultaneous sonication/plating (i.e., sono-electrodeposition) have been shown to enhance the fractality of nanometals deposited on the surface [32], but detailed studies of batch-to-batch variability using this technique have not been explored to the best of our knowledge. Further studies are required to understand the underlying mechanism(s) of this variability in system capacitance and for the development of strategies to improve the repeatability. 

The results shown here are in line with Avinash et al. [4], noting the high degree of variation for laser-ablated graphene. In accordance, we suggest that studies are needed on standardizing laser-processing equipment techniques and experimental parameters for micro-device fabrication. Additional factors of importance are the orientation of electrode scribing, which was shown to be important in the study by Behrent et al. [26]. The data from this study are available (open access) at Zenodo; see data availability statement. The data files in the repository contain all experimental metadata and equipment/instrument metadata. One of the limitations of the data are the use of a relatively high scan rate (200 mV/s), which may induce some Ohmic drop. No corrections were applied to account for uncompensated resistance, which is a consideration for follow-up studies. In addition, we have performed other analyses not represented in this body of work (e.g., Raman microscopy, electrokinetic analysis, atomic force microscopy); results and additional datasets will be appended to the Zenodo repository as they are available (in raw data format). 

## 4. Conclusions

Laser-inscribed graphene (LIG) is an emerging material for micro-electronic applications and is being used to develop supercapacitors, soft actuators, triboelectric generators, and sensors. The fabrication technique is simple, yet the batch-to-batch variation of LIG quality is not well documented in the literature. For the first time, we conducted a study to characterize batch-to-batch variation in the manufacturing of LIG electrodes for applications in electrochemical sensing. Numerous batches of 36 LIG electrodes were synthesized using a CO_2_ laser system and polyimide film. The LIG material was characterized using goniometry in various solutions, stereomicroscopy, open circuit potentiometry, and cyclic voltammetry (CV). The contact angle and average OCP for LIG electrodes varied by approximately 5%, but the variability was significantly higher for LIG electrodes metallized with platinum. Contact angle did not vary across batches, but the type of liquid tested did have a significant effect on mean contact angle. Although each buffer is commonly used in sensor development/testing, a number of the compounds are zwitterionic or emulsifying agents that are known to alter wettability. Characterization of the hydrophobicity in these various buffers provides key insights into the surface charge of the graphitized/metallized materials. When LIG sensor chips were fabricated (working, counter, and reference electrode composed of LIG), the peak current at 200mV/sec decreased significantly, although the batch-to-batch variation was similar (5–10%). After metallization of LIG with nanoplatinum (nPt), anodic/cathodic current, and area between CV curve increased significantly. However, the batch variation increased to 20% within groups and up to 40% across groups. Taken together, these results indicate that metallization of LIG with nPt improves performance but comes at the expense of increased batch variability. This study provides important insight into the variation of material properties, and we note that additional studies are needed to understand the mechanisms with the aim of improving quality control in LIG micro-device scaling. The dataset is available via an open access repository for additional analysis.

## Data Availability

Cyclic voltammetry data are available in an open access repository at Zenodo: https://zenodo.org/communities/qclig/records?q=&l=list&p=1&s=10&sort=newest.

## Supplementary Materials

The following supporting information can be downloaded at: https://www.mdpi.com/article/10.3390/mi15070874/s1, Table S1. Contact angle for non-modified LIG in various solutions measured with a Droplet lab DROPOMETER-M; Table S2. Features from electrochemistry dataset for this study; Table S3. Average peak current and potential for all LIG tested; Figure S1. Process of laser induced graphitization of polyimide; Figure S2. Heat map for laser fabrication settings of LIG based on study by Behrent et al. [26]; Figure S3. Open circuit potential measured in bicarbonate buffer at room temperature; Figure S4. *p*-value heat map of conditioning CV indicating significant testing of iop relative to the 1st scan; Figure S5. Raw cyclic voltammograms at 200 mV/s (ferri/ferrocyanide with potassium chloride as electrolyte); Figure S6. Five treatments were designed to determine the ideal nPt electrodeposition condition on both a single electrode (control) and biochip system.
