# Supplementary material for: Batch-to-Batch Variation in Laser-Inscribed Graphene (LIG) Electrodes for Electrochemical Sensing
Source: Micromachines (Basel). 2024 Jun 30;15(7):874. doi: 10.3390/mi15070874 (PMC11279040; doi:10.3390/mi15070874)
Supplement: Supplementary file 1 [file micromachines-15-00874-s001.zip › micromachines-3022707-supplementary.pdf]

micromachines

Supplementary Materials

Half cell constant (E_0) determination

For a general redox couple:

$$O + e^- \leftrightarrow R \qquad \textbf{(Equation S1)}$$

Applying Nernst theorem, the equation describing open circuit potential (OCP) is then:

$$E = E^0 + \frac{RT}{nF} ln\left(\frac{[O]}{[R]}\right) \qquad \textbf{(Equation S2)}$$

For equimolar concentrations of oxidized and reduced species:

$$E = E^0 \qquad \textbf{(Equation S3)}$$

For the electrochemical couple ferri/ferrocyanide used in this study:

$$K_3Fe(CN)_6 + e^- \leftrightarrow K_4Fe(CN)_6 \qquad \textbf{(Equation S4)}$$

then:

$$E = E^0 + \frac{RT}{nF} ln\left(\frac{[K_3Fe(CN)_6]}{[K_4Fe(CN)_6]}\right) \qquad \textbf{(Equation S5)}$$

at room temperature (25°C), the constant term (RT/nF) is:

$$\frac{RT}{nF} = \frac{\left(8.1345\ ^{J}/_{mol-K}\right)*(298.15K)}{(e^-)*\left(96485.3\ ^{C}/_{mol-e^-}\right)} = 0.0241V \qquad \textbf{(Equation S6)}$$

If OCP is monitored at room temperature (25°C), the ratio of ferricyanide to ferrocyanide is:

$$\frac{[O]}{[R]} = \left(\frac{[K_3Fe(CN)_6]}{[K_4Fe(CN)_6]}\right) = e^{(E-E_0)/_{0.0241V}} \qquad \textbf{(Equation S7)}$$

Table S1. Contact angle for non-modified LIG in various solutions measured with a Droplet lab DROPOMETER-M. For all experiments, a 5 µl aliquot of solution was pipetted on the working area of LIG electrode and contact mode recorded in sessile droplet mode. All measurements recorded at 25°C. pH calibration was Nersntian (E_0= 406 mV; calibration slope = 58.3 mV/log-[H^+]).

Solution Type	pH	Average contact angle for LIG [deg.]	Average contact angle for nPt-LIG [deg.]
Deionized water (DI)	7.8	58.6 ± 1.5	77.8 ± 4.1
Sodium bicarbonate (isotonic) (PubChem ID: 516892)	8.1	59.3 ± 2.6	78.2 ± 4.2
MES buffer (PubChem ID: 16218417)	6.2	56.9 ± 3.6	65.6 ± 1.4
Tris buffer (PubChem ID: 6503)	6.6	57.5 ± 0.9	62.6 ± 1.5
HEPES buffer (PubChem ID: 23831)	7.5	61.4 ± 2.0	60.7 ± 1.4

Table S2. Features from electrochemistry dataset for this study (available at Zenodo: https://zenodo.org/communities/qclig/records?q=&l=list&p=1&s=10&sort=newest). All electrodes tested in tested at 200mV/sec in redox solution *. The total features in the open-source dataset is 210,450.

Solution	Number of datapoints
Bare (non-modified) LIG single electrode	85,323
nPt-coated LIG single electrode	15,950
Bare (non-modified) LIG sensor chip	124,410
nPt-coated LIG sensor chip	12,760

* Redox solution = 2.5 mM ferricyanide + 2.5 mM ferrocyanide + 100 mM potassium chloride.

Table S3. Average peak current and potential for all LIG tested (available at Zenodo: https://zenodo.org/communities/qclig/records?q=&l=list&p=1&s=10&sort=newest).

Electrode Type	I_{op} [µA]	E_{op} [mV]	I_{rp} [µA]	E_{op} [mV]
LIG	214 ± 27	421 ± 42	-218 ± 23	31 ± 45
nPt-LIG	388 ± 119	377 ± 41	-425 ± 139	-346 ± 268
LIG chip	114 ± 49	375 ± 31	-125 ± 42	-343 ± 141
nPt-LIG chip	359 ± 140	364 ± 26	-370 ± 128	-397 ± 199

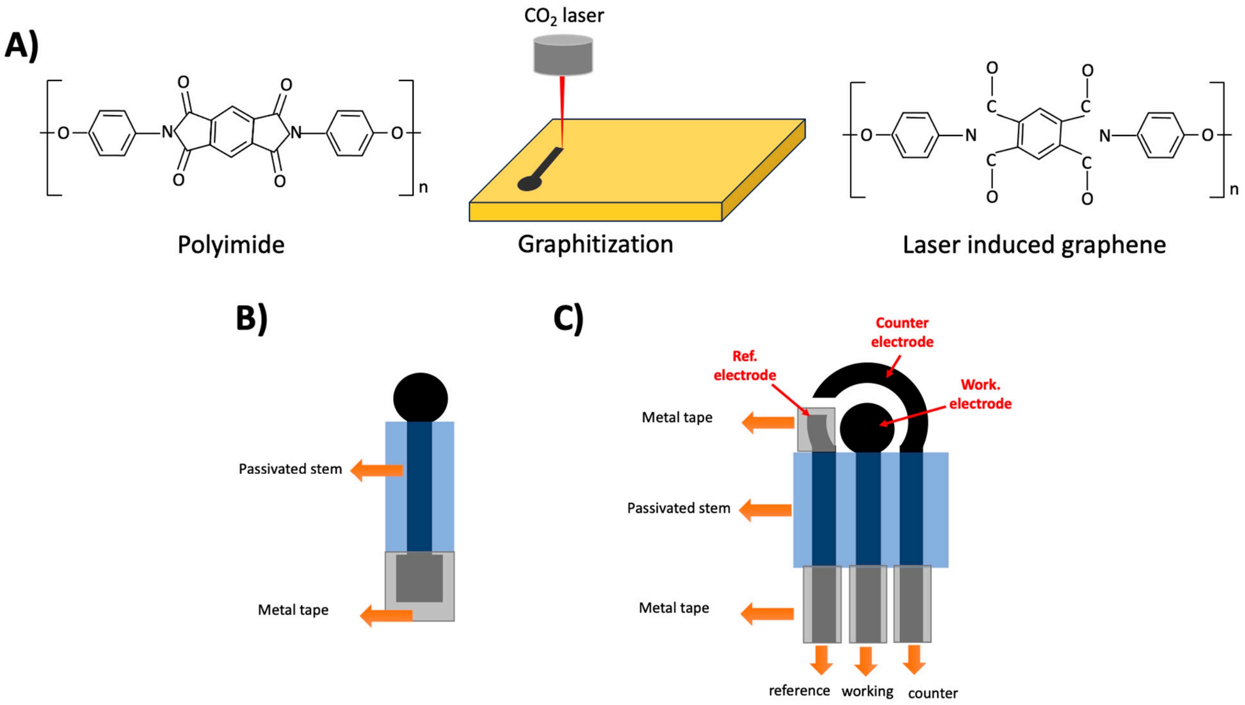

Figure S1. A) Process of laser induced graphitiziation of polyimide. The process converty polyimide (an sp3 form of carbon) to a heterogenous mixture of graphiitizied material (sp^2-hybrized carbon). Schematic of **B)** single LIG working electrode and **C)** three electrode LIG sensor chip. The passivated stem position and bonding pad of each electrode is indicated in blue and grey, repsectively.

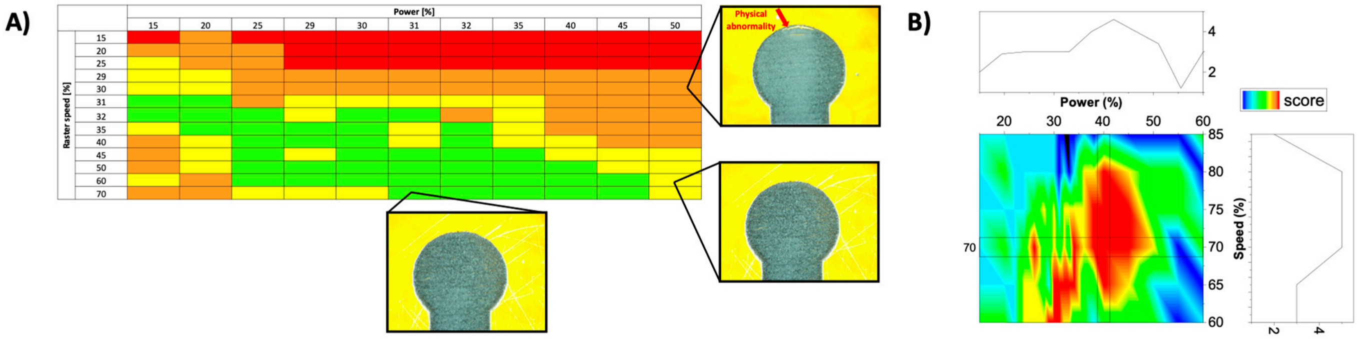

Figure S2. Heat map for laser fabrication settings of LIG based on study by Behrent et al [26]. **A)** Stereomicroscope imaging of LIG identified physical abnormalities for non-optimal settings (indicated in red). Sub-optimal laser settings (indicated by yellow or orange) did not reveal any physical defects, but additional electrochemical analysis indicated that the electrodes were not ideal. **B)** Combination of stereoscopic inspection, open circuit potential, and cyclic voltammetry at 200mV/sec were used to analyze single LIG working electrodes. A Ag/AgCl reference electrode and Pt wire were used as reference and counter for all voltammetry in ferro/ferricyanide (with 100mM KCl). Results from microscopy (pass/fail), potentiometry (measurable signal), and voltammetry (peak oxidation potential for quasi-reversible plot) were compiled by developing a equal-weighted score. Heat map of scoring system for laser settings where: low scores (1, blue) represent material that is cracked and/or has redox peaks. High scores (5, red) represent high redox peaks and no visible damage. Details of scoring system are in Tang [33].

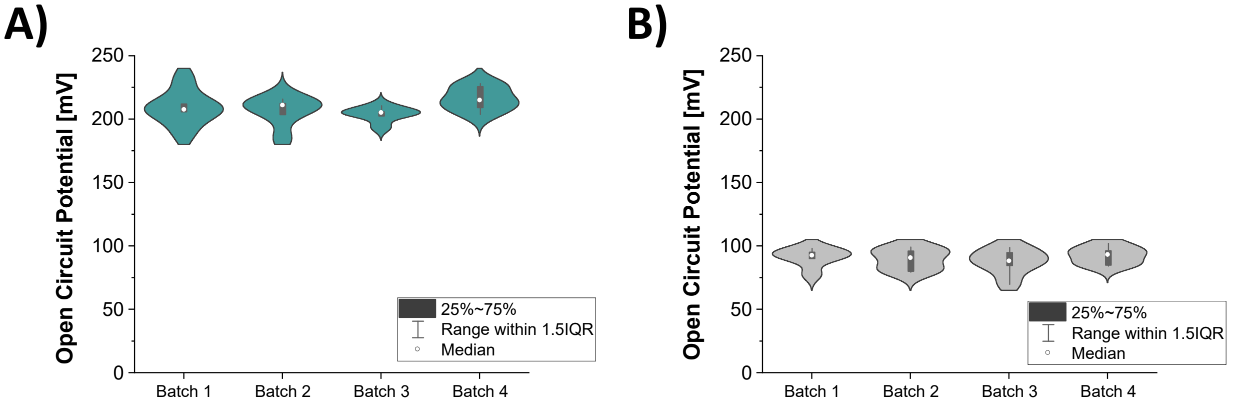

Figure S3. Open circuit potential measured in bicarbonate buffer at room temperature. Average OCP was calculated based on 120s of chronopotentiometry at a data acquisition rate 10kHz. **A)** Non-modified (bare) LIG single electrode, **B)** nPt-coated LIG single electrode. For all experiments, 36 electrodes were tested.

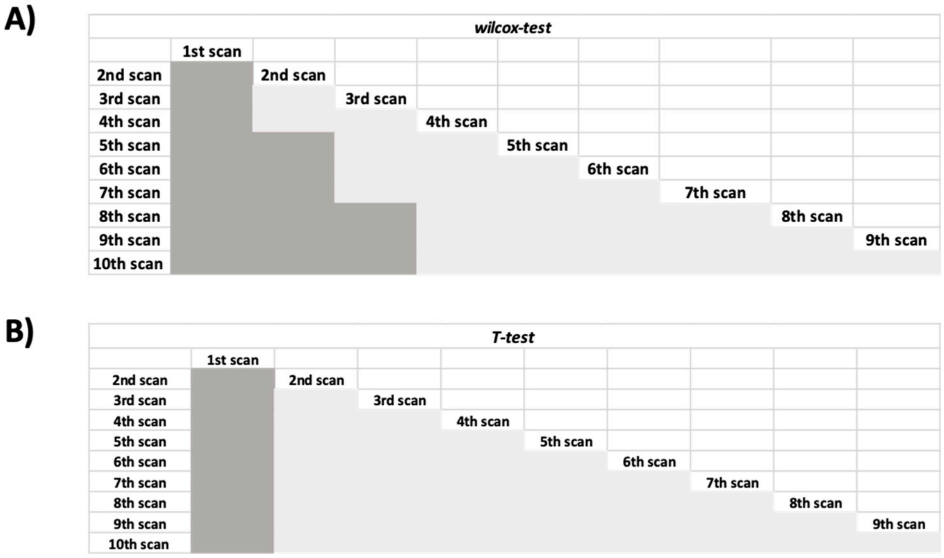

Figure S4. P-value heat map of conditioning CV indicating significant testing of i_{op} relative to the 1st scan. Difference tests of i_{op} from each scan were analyzed for a) single LIG electrode, and b) LIG biochip. Shapiro tests indicated that data in panel a were not normally distributed, while data in panel b were normal. In panel a, Wilcox test (not normally distributed) were used to create a pair-wise heat map. In panel b, t-test (normally distributed) were used for analysis of biochip data. Dark grey blocks indicate p-values smaller than 0.05 (significantly different), light grey blocks indicate p-values greater than 0.05 (not significantly different).

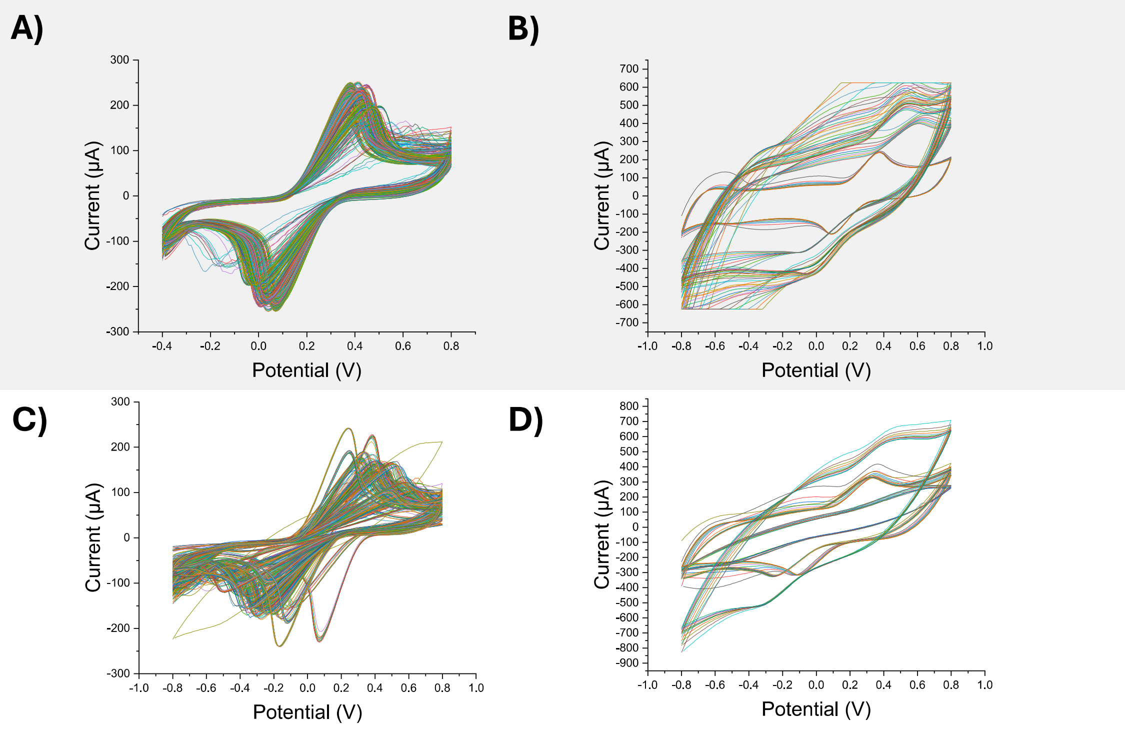

Figure S5. Raw cyclic voltammograms at 200mV/sec (ferri/ferrocyanide with potassium chloride as electrolyte). **A)** Single LIG electrode (n=360); **B)** nPt-LIG single electrode (n=50); **C)** LIG sensor chip (n=390); **D)** nPt-LIG sensor chip (n=40).

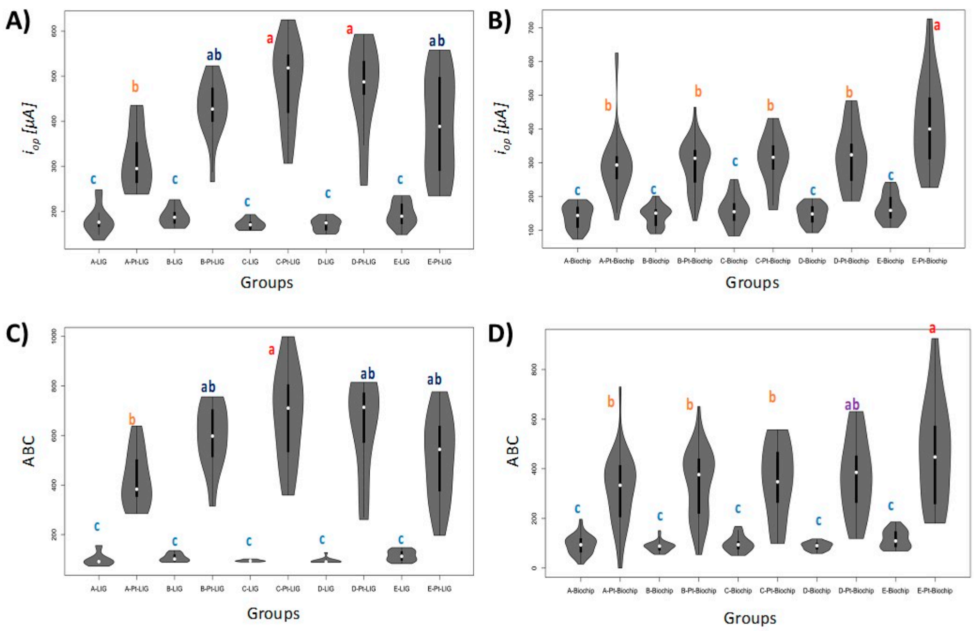

Figure S6. Five treatments were designed to determine the ideal nPt electrodeposition condition on both a single electrode (control) and biochip system. Violin plots of i_{op} and area between curve depicting mean data and variance (at least 50 electrodes were analyzed in each batch). White dots represent median value, black boxes range from the lower to the upper quartile, whiskers present the variability outside upper and lower quantile, and the shape of violin plot indicates the data density. **A)** peak current for bare LIG and nPt-LIG in different treatments; **B)** peak current for bare LIG biochip and nPt-biochip; **C)** area between curve for bare LIG and nPt-LIG; **D)** area between curve for bare LIG biochip and nPt-biochip. Lower case letters represent statistically distinct groups based on pairwise t test at α=0.05.
